# County Asylums as War Hospitals

**Published:** 1915-08-14

**Authors:** 


### COUNTY ASYLUMS AS WAR HOSPITALS.

### By a Medical Organiser.

A notable addition to the accommodation for our wounded was made when the War Office arranged with the authorities concerned to take over certain of the asylums as war hospitals. This decision placed at the disposal of the Army several thousand beds in hospitals, some of them of the most recent type, all of them near to it. Here was little necessity for improvisation. To hand were all the conveniences required; good administrative blocks, light, large wards, ample sanitary accommodation, and beautiful grounds for the convalescent.

The Board of Control, the asylums committees of the various authorities concerned, and the staffs of the asylums gave ready, willing, and patriotic support to the scheme; in a very short space of time the original inmates were distributed amongst the asylums not to be utilised for wounded, and the war hospitals were organised and in full work.

In broad outline, the system followed has been to give the superintendent of the asylum a temporary commission in the R.A.M.C., to appoint him administrator, and to place under him a resident medical staff, composed in part of his original assistants in the asylum and in part of medical men from outside who have volunteered for the work. Since it was not desired nor desirable to employ in these residential staffs young, recently-qualified men who would be useful nearer the Front, these organisations have given an opportunity to many medical men to render personal service to their country which otherwise might not have offered. The response to the call has been noble. Amongst those who have answered it are men in their prime, who have abandoned for the time being lucrative surgical practices, men who have left expanding specialist practices, cheerfully chancing the risk of the loss of the fruit of the labour and waiting that built them up, and men who had grown grey in general practice and had retired to take their ease. And these men are not offering a sham sacrifice, with a few thousand a year pay and honour and the limelight to offset what sacrifice there be, but are in the background, on low pay, performing the subordinate duty of house surgeon to men with whom many of them can compare in qualifications and abilities. They can neither expect nor gain anything but the satisfaction of feeling that they have done for their country that which it lay to their hand to do. All honour to them!

Next in order—superior to the resident staff, but under the administrative control of the administrator—come the visiting staff. This is composed of surgeons and physicians, among the most able and best known in the neighbourhood of the asylum. Working with the resident staff, the men of the visiting staff are responsible for the surgical and medical treatment in every department of medical science. Possessed of supreme skill, and with every modern apparatus at their disposal, the results obtained by this co-operation are as good as can be desired. Glad and proud to do this work for their country, the men of the visiting staff are working from a sense of duty to their country, not for gain; the pay is a pound a visit, and a visit may last half the day and include half-a-dozen major operations, or entail a few hours in the $x$-ray room or an equal time in the eye department. The nursing staff is composed of the asylum attendants, male and female, supplemented, as to the male half, by men of the St. John Ambulance and other men enlisted for the work, as to the female half by specially appointed trained nurses. The male staff has been attested and enlisted on special terms as soldiers for the duration of the war. The female staff is free to leave on due notice, and is under the command of a matron; in most cases a special appointment. These subordinate staffs are doing good work, and make up in zeal for the lack of training of many amongst them. The administrators have a hard and responsible task. It is for each of them to ensure harmonious working in a large and suddenly organised machine under conditions of which they have not had previous experience. The success of these ventures is proof of the tact and ability that the seniors of the Asylum Service acquire in the course of their duties.

The various corporations that control the asylums taken over, by means of their asylums committee, undertake the finance, structural alterations, and provisioning of the war hospitals. The work they perform is most valuable, since it takes the place of the work done by the A.S.C and the R.E., and so frees these important and hard-worked departments of the Army from any necessity to divert their strength into this direction. The war hospitals are considered as central hospitals, and have as a rule hospitals managed by Voluntary Aid Detachments affiliated to them. These affiliated hospitals receive such cases from the central hospital as the surgical and medical staffs may select, take care of them till fit for discharge, and so relieve the central hospital.

These notes, necessarily brief, of the war work of some of our great institutions, make conspicuous the fact that the members of the great profession of medicine are doing their duty as patriots right well, and are setting an example to the nation worthy of the traditions of that profession, and that this lead is being nobly followed by the institutional subordinates who have been habituated to work with them—a record of which all concerned may be proud.

